# Skin Diseases and Syphilis

**Published:** 1893-07

**Authors:** 


### *SELECTIONS AND ABSTRACTS.*

---

### SKIN DISEASES AND SYPHILIS.

---

EDITED BY DR. M. B. HUTCHINS.

---

#### TREATMENT OF SYPHILIS.

Dr. L. Brocq, of Paris, in letter to June *Journal of Cutaneous and Genito-Urinary Diseases,* mentions Fournier's method of treating syphilis. He gives to a patient coming early in the disease a course of proto-iodide of mercury for two months. Then the treatment is suspended for a month or six weeks. No matter what may have occurred in this time he gives the proto-iodide for another six weeks, then suspends for two or three months. He continues this intermittent treatment until the third year. Then comes iodide of potassium in periods of a month to six weeks, the intervals of rest growing longer with the time since the beginning of the disease. Others, notably Diday of Lyons, insist that treatment must only be given while there are visible manifestations of the disease. The latter believe that mercury has no preventive action—no power to prevent syphilitic manifestations.

Brocq inclines to believe that the treatment should be persisted in, as the doses of the present day are not injurious and may prevent severe accidents.

The regular, intermittent, plan has the serious objection that nine out of ten syphilitics would fail to follow it. The following of the plan of treating them only when manifestations are visible would be easier, because the patient then has a strong incentive to take treatment. This latter method should suffice, for all who have had experience in this disease know how easily proper treatment controls all symptoms and renders the disease innocuous to the patient or to others.

### Experiments in the Prevention of Syphilis and Chancroid.

The above quoted journal has a reference to efforts made to prevent the "taking" of inoculations with the virus from the above diseases. The experiments were based upon the facts that the secretions from chancroid and from recent syphilides were alkaline, and that soft chancres and especially primary syphilitic ulcers were rare inside the prepuce because the normal secretion there was acid. So the experimenter thought of a mixture which would be acid, and also strongly disinfectant, without being a caustic agent.

To a three per cent. solution of peroxide of hydrogen he added proportions of one-half per cent. of nitric acid (?). The secretion of chancroid was mixed with one to four drops of the compound and inoculated upon the well cleansed forearm. Fourteen out of fifteen of these inoculations did not "take."

The same persons were inoculated with chancre secretion alone, without the mixture, and all "took."

A person inoculated with chancre secretion mixed with the peroxide of hydrogen rendered alkaline with potash lye got a chancre. Inoculations with the secretion from "primary" syphilitic ulcers, or from syphilitic warts mixed with the original acid compound failed.

Two healthy physicians allowed themselves inoculated as above, no occurrence of syphilis followed.

The reference closes with the remark that it will do more to abate the evil results of prostitution than anything else if the use of this mixture just before coitus will protect from infection.

It might be well to have a police regulation requiring the demimonde to syringe with this mixture before entertaining a visitor.

---

### Extensive Symmetrical Herpes Zoster ("Shingles") with Carbuncles as Sequelæ.

This case was reported by Wm. S. Sharpe in the March *British Journal of Dermatology.* Man of sixty-four struck on the head and the middle of the back by an engine in November, 1886. March, 1889, an attack of herpes zoster in area of distribution of the seventh, eighth and ninth dorsal nerves on each side. Remaining dorsal and the lumbar and sacral nerve areas subsequently involved, an interval of two or three days between affection of each nerve and the following. The right side usually involved a few days before the left corresponding. When the lumbar region had become affected small carbuncles began to appear on the site of every large group of vesicles beginning in the first and involving the others in the order of their duration. The longest diameter of these carbuncles was in the course of the nerve. Albumin and sugar not present in the urine. The writer remarks upon the rarity of symmetrical zoster. The previous injury to the spine was considered the cause of the skin trouble. He does not think the carbuncles were due to infection. It appears to have been a case in which the nerve involvement was so intense that a much larger, a continuous, area was involved than we see in herpes zoster taking on the gangrenous process.

---

### TREATMENT OF ECZEMA WITH "ZINC-OIL."

Richard Drews (*Wien. Med. Wochenschr*, 1892, No. 51) has found the mixture of oxide of zinc and olive oil, recommended by Lassar, very useful in eczema of children. The thickness of the paste depends upon the quantity of zinc oxide. Dr. Drews employs the following proportion in eczema intertrigo, after the surface is cleansed, with strips of cloth :

Zinc oxide, ℥ 3.

Olive oil, ℥ 5.

This zinc oil may also be used to protect the skin from maceration or irritation of antiseptic dressings.—*Monatshefte für Prak. Derm.*, April, 1893.

This preparation can be easily made by country doctors. They can use cotton seed oil or other bland oil if they cannot get the olive oil. City doctors do not always get olive oil dispensed, but cotton seed oil seems to do as well. The zinc oil may sometimes be too strong (there are people whose skin does not seem able to bear zinc oxide). Corn starch might be substituted for part of the zinc, and the thickness of the mixture thus preserved.

### BARBER SHOP HYGIENE.

*Monatshefte für Praktische Dermatologie* of April 15, 1893, reports a discussion of this subject which occurred in a meeting of the Berlin Dermatological Society. It has long been known that the barber shop may be the source from which a person gets certain skin diseases. The barber may not have the disease but some customer has left the germs in the shaving brush, towels (possibly the razor), the powder "puff" as well as the common comb and brush. Any contagious disease might be thus communicated. To avoid chances of contagion a customer might keep a full outfit of his own at the barber shop and see that the barber *disinfects his hands.*

---

### ERYSIPELAS AND ECZEMA.

The most striking point of difference between these is the shining, glossy, tense appearance of an erysipelas. An erythematous eczema, the one most likely to be called erysipelas, may have some general resemblance to erysipelas, as when on the face, but the surface is faintly roughened and scaly from the first, less bright, borders ill defined.

